# PIK3CA mutation-driven immune signature as a prognostic marker for evaluating the tumor immune microenvironment and therapeutic response in breast cancer

**DOI:** 10.1007/s00432-024-05626-4

**Published:** 2024-03-11

**Authors:** Xueting Ren, Hanxiao Cui, Luyao Dai, Lidan Chang, Dandan Liu, Wenyu Yan, Xuyan Zhao, Huafeng Kang, Xiaobin Ma

**Affiliations:** Department of Oncology, The Second Affiliated Hospital of Xi’an Jiaotong University, Xi’an, Shaanxi China

**Keywords:** Breast cancer, PIK3CA mutation, Tumor immune microenvironment, Prognosis, Immunotherapy

## Abstract

**Purpose:**

Gene mutations drive tumor immune microenvironment (TIME) heterogeneity, in turn affecting prognosis and immunotherapy efficacy. PIK3CA is the most frequently mutated gene in breast cancer (BC), yet its relevance to BC prognosis remains controversial. Herein, we sought to determine the impact of PIK3CA mutation-driven immune genes (PDIGs) on BC prognosis in relation to TIME heterogeneity.

**Methods:**

PIK3CA mutation characteristics were compared and verified between the TCGA-BRCA dataset and a patient cohort from our hospital. PIK3CA mutation-driven differentially expressed genes were identified for consensus clustering and weighted gene co-expression network analysis to select the modules most relevant to the immune subtype. Thereafter, the two were intersected to obtain PDIGs. Univariate Cox, LASSO, and multivariate Cox regression analyses were sequentially performed on PDIGs to obtain a PIK3CA mutation-driven immune signature (PDIS), which was then validated using the Gene Expression Omnibus (GEO) database. Differences in functional enrichment, mutation landscape, immune infiltration, checkpoint gene expression, and drug response were compared between different risk groups.

**Results:**

PIK3CA mutation frequencies in the TCGA and validation cohorts were 34.49% and 40.83%, respectively. PIK3CA mutants were significantly associated with ER, PR, and molecular BC subtypes in our hospital cohort. The PDIS allowed for effective risk stratification and exhibited prognostic power in TCGA and GEO sets. The low-risk patients exhibited greater immune infiltration, higher expression of common immune checkpoint factors, and lower scores for tumor immune dysfunction and exclusion.

**Conclusion:**

The PDIS can be used as an effective prognostic model for predicting immunotherapy response to guide clinical decision-making.

**Supplementary Information:**

The online version contains supplementary material available at 10.1007/s00432-024-05626-4.

## Introduction

In 2020, female breast cancer (BC) was the most common malignant tumor worldwide and the leading cause of cancer-related death in women (Sung et al. 2021). It is estimated that 287,850 new cases of female BC and 43,250 deaths will occur in 2022 in the US alone (Siegel and Miller 2022). In recent years, surgery, radiotherapy, chemotherapy, immunotherapy, endocrine, and targeted therapy have been widely applied for the treatment of this heterogeneous malignancy, leading to significant progress. However, the global burden of BC remains considerable, necessitating the early identification of high-risk patients and precision medicine-inspired solutions for improved prognosis.

Phosphatidylinositol 3-kinases (PI3Ks) phosphorylate the third hydroxyl of the phosphatidylinositol ring, and they can be classified into three types based on structure (I, II, and III) (Mosele et al. 2020). Type I are the most widely studied, acting in various important biological processes, such as cell growth, proliferation, metabolism, and migration through the PI3K/AKT/mTOR pathway (Samuels et al. 2005). The PIK3CA gene encodes the catalytic subunit of class IA PI3Ks (p110α) (Zardavas et al. 2014). Mutation of PIK3CA can lead to abnormal catalytic activity of PI3Ks and, consequently, promote carcinogenesis in various tissues (Herberts et al. 2020; Hu et al. 2021; Jin et al. 2021; Ugai et al. 2021). PIK3CA somatic mutations occur in approximately 30% of BC patients, being more common in hormone receptor (HR)-positive tumors (Lv et al. 2020). Approximately 80% of PIK3CA mutations occur in the helical and kinase domains, with E542K and E545K in exon 9 as well as H1047R and H1047L in exon 20 as the most common variants (Dirican et al. 2016). In the past few years, PIK3CA has emerged as a promising target for BC treatment, with alpelisib and fulvestrant receiving approval for the treatment of PIK3CA-mutated, endocrine-resistant HR+/HER2− locally-advanced or metastatic BC (De Mattos-Arruda 2020). Moreover, several large clinical trials have reported an association of PIK3CA mutations with favorable prognosis and clinicopathological BC features (Kalinsky et al. 2009; Loi et al. 2013). Therefore, further study of the differences in gene expression between PIK3CA-mutated and wild-type BC should provide valuable insight for predicting prognosis and guiding clinical decision-making.

In light of the impact that the tumor immune microenvironment (TIME) has on tumorigenesis and metastasis, there has been increasing interest in immunotherapy for BC. Immune checkpoint inhibitors (ICIs) targeting cytotoxic T lymphocyte-associated protein 4 (CTLA4), programmed cell death-1 (PD-1), and programmed cell death ligand-1 (PD-L1) have shown promising efficacy in certain tumor types, leading to their entry into the clinic (Gaynor et al. 2022). The immunoregulatory effects of CTLA4 antagonists tremelimumab and ipilimumab have been confirmed in small-scale BC cohorts (Zhu et al. 2021). Clinical trials of CTLA4 antagonist monotherapy or in combination with other immunomodulators are ongoing, with the clinical benefits of CTLA4 inhibition in BC expected to be confirmed in the future. Anti-PD-1/PD-L1 immunotherapy confers a survival benefit to some metastatic triple-negative BC patients (Adams et al. 2019; Schmid et al. 2017). The TIME, which is composed of infiltrating immune cells and various other cell types, is strongly correlated with tumor progression and ICI response (Miller et al. 2021; Oliver et al. 2019; Taylor et al. 2017). Tumor-infiltrating lymphocytes (TILs) are major indicators of immune infiltration in the TIME and can inhibit tumor growth (Bagbudar et al. 2022). Enhancing cytotoxic T cell responses may suppress tumor growth and improve patients survival (Rupp et al. 2022). Studies have shown that BC tumors with a higher proportion of TILs are more sensitive to chemotherapy, radiotherapy, as well as immunotherapy, leading to improved prognosis (Adams et al. 2014; Denkert et al. 2018). Some studies have reported the effect of different mutations on ICI response (Chen et al. 2021a; Collins et al. 2022). Currently, the main challenge of immunotherapy remains the exploration of reliable indicators to predict its potential efficacy.

Hence, we explored the mutation characteristics of PIK3CA in BC samples from the Cancer Genome Atlas Database (TCGA, https://portal.gdc.cancer.gov/) and our institution. Subsequently, a PIK3CA mutation-driven immune signature (PDIS) was developed based on TCGA data, and we validated its capacity for BC patient risk stratification using the Gene Expression Omnibus database (GEO, https://www.ncbi.nlm.nih.gov/geo/). The signature presented herein may be employed to evaluate the TIME, optimize clinical benefit, and predict patient prognosis.

## Materials and methods

### Data collection and processing

Somatic mutation profile, RNA-seq data of BC and normal samples (FPKM-normalized format), as well as corresponding clinical features (including age, survival time, survival status, TNM stage, pathological stage, and ER/PR/HER2 receptor status) were collected from TCGA. Patients without survival information were excluded from subsequent analyses. We implemented the following inclusion criteria for cohorts from the GEO database: (1) samples were derived from human BC; (2) RNA-seq expression data was available; (3) clinical and prognostic information of patients was available; (4) the number of samples was greater than 50.

### PIK3CA mutation analysis of BC samples from local hospital

To validate the results of mutation analysis in TCGA, we obtained surgically resected female BC samples from the Department of Oncology at Second Affiliated Hospital of Xi’an Jiaotong University obtained from January 2019 to July 2022. Screening criteria included: (1) no preoperative neoadjuvant therapy, (2) postoperative pathologically confirmed BC, and (3) pathological tissues eligible for molecular typing. This study was approved by the Ethics Committee of the Second Affiliated Hospital of Xi’an Jiaotong University. The surgical procedure was performed in accordance with the Declaration of Helsinki. All patients signed an informed consent form. We employed the allele-specific polymerase chain reaction (AS-PCR) method for PIK3CA mutation detection in patient samples. DNA was extracted from formalin-fixed, paraffin-embedded (FFPE) samples using the QIAamp DNA FFPE Tissue Kit (Qiagen, Cat No./ID: 56,404). Five hotspot mutations of PIK3CA (H1047R, H1047L, E542K, E545K, E545D) were then examined as per the manufacturer’s instructions using the Human PI3K Gene Mutation Fluorescence PCR diagnostic kit (Amoy Diagnostics, Xiamen, China) on an SLAN-96S fluorescent quantitative PCR instrument. Based on the obtained Ct values, we divided samples into negative, weakly positive, and strongly positive, with the latter two considered to harbor mutations.

### Somatic mutation analysis and clinical validation

BC mutation data, including mutated genes, mutation types, and mutation sites, were downloaded from TCGA “Masked Somatic Mutation” database. The waterfall function in the “maftools” package was applied to obtain the mutation landscape of the TCGA-BRCA cohort. To understand the distribution of PIK3CA mutations in BC and their correlation with clinicopathological factors, PIK3CA mutation samples and related information were extracted from the downloaded mutation data, with clinical samples obtained from our local hospital used for comparison and validation. Correlations between PIK3CA mutations and clinical variables were analyzed using Pearson’s Chi-square test or Fisher’s exact test (Shao et al. 2021).

### Identification of differentially expressed genes (DEGs) driven by PIK3CA mutations

RNA-seq data of PIK3CA^MUT^ and PIK3CA^WT^ patient tumor samples were extracted. After normalization, the “edgeR” package was further used to identify DEGs driven by PIK3CA mutations in BC patients. The screening criteria were set as the adjusted *P* < 0.05, log_2_|fold change (FC)|> 0.

### Consensus clustering

Single-sample gene set enrichment analysis (ssGSEA) was used to analyze the infiltration of 22 immune cell types and the activity of seven immune-related pathways in tumor tissue samples (Hänzelmann et al. 2013). Based on the extent of immune infiltration, we applied the “ConsensusClusterPlus” package for consensus clustering to divide BC patients into two groups of high- and low-immune activity (Seiler et al. 2010).

### Weighted gene co-expression network analysis

The “WGCNA” package was used to construct a gene co-expression network to aggregate genes with highly correlated expression and identify gene modules closely related to immune subtypes (Liu et al. 2022). Firstly, a similarity matrix was constructed based on expression data, and the Pearson correlation coefficient was calculated to evaluate the similarity between genes (Langfelder and Horvath 2008). An adjacency matrix was then constructed based on the above matrix. The formula was as follows: aij = power (sij, β) =|sij|^β^, where ajj represents the correlation strength between gene i and gene j, sij is the Pearson correlation coefficient between the two genes, and β is the soft threshold (power) (Zhang and Horvath 2005). By constructing a topological overlap matrix (TOM), hierarchical clustering analysis of genes was performed, and genes with similar expression patterns were divided into gene modules using dynamic branch cut methods (Tian et al. 2020). Each module contained at least 200 genes, and modules with correlations greater than or equal to 0.75 were merged. Finally, the Pearson correlation coefficient between the MEs of each module and the clinical traits was calculated, and the module most related to the immune subtypes were screened for subsequent analysis. Module eigengene (ME, The first principal component in each module) described the overall level of gene expression in the Module. Module membership (MM, Correlation of all gene expression profiles with eigengene of this module) and gene significance (GS, The absolute value of the correlation between genes and phenotypic traits) were used to evaluate the correlation between genes in the module and the module itself, along with the correlation with the corresponding traits of the module (Tian, et al. 2020).

### Construction and validation of PDIS

A Venn diagram was used to intersect genes driven by the PIK3CA mutation and the module most relevant to immunity. The obtained genes were termed PIK3CA mutation-driven immune genes (PDIGs). Univariate Cox regression analysis was performed on the obtained PDIGs to screen those related to overall survival (OS) of BC patients. Then, using the “glmnet” and “survival” packages, least absolute shrinkage and selection operator (LASSO) was conducted on the above genes. By controlling the penalty coefficient λ, the coefficients of some genes less related to prognosis were compressed to 0, and the coefficients of genes significantly related to prognosis were retained greater than 0. Then a multivariate model was constructed to further confirm the genes independently associated with prognosis. Risk score = h(t, X) = h_0_(t) × e^Ʃ (coefi*Expri)^, where h0(t), coefi, and Expri are the constant, regression coefficient, and gene expression level, respectively (Ren et al. 2022). Patients in TCGA training and GEO validation cohorts were divided into high- and low-risk groups according to the corresponding median risk score, of which the high-risk patients are those with PDIS score above the median and the low-risk patients are those with PDIS score below the median. Kaplan–Meier (KM) survival analysis was performed using the “survival” and “survminer” packages to explore the capacity of PDIS and PIK3CA mutation status or regions to differentiate prognosis between risk groups. Other recently published prognostic models of BC were retrieved from PubMed (https://pubmed.ncbi.nlm.nih.gov/). The “survivalROC” package was used to plot the receiver operating characteristic curves (ROC), whereafter the area under the ROC curve (AUC) and C-index were used to evaluate and compare the predictive validity of multiple models.

### Establishment and verification of predictive nomogram

Combining with risk score and other clinicopathological features, R packages “rms” and “regplot” were used to construct a nomogram to quantitatively predict the survival rate of BC patients. Then, using the calibration function and “survivalROC” package, calibration and ROC curves of 1-, 3-, 5-year survival were drawn to verify the predictive ability of the nomogram. Decision curve analysis (DCA) was performed on different prognostic factors using the “ggDCA” package.

### Functional enrichment analysis

DEGs between the high- and low-risk groups were screened with log_2_|FC|> 1 and an adjusted *P* < 0.05 as the standard. The “clusterProfiler” package was then used to perform Gene Ontology (GO) and Kyoto Encyclopedia of Genes and Genomes (KEGG) pathway enrichment analysis to explore the potential function of DEGs (Yu et al. 2012). Next, gene set enrichment analysis (GSEA) was used to explore the signaling pathways associated with different risk subgroups.

### Mutation landscape and TMB analysis

The “maftools” package was used to visualize mutations in the high- and low-risk groups as well as to compare the two groups in this regard. The total number of mutations per megabase in each sample was calculated to obtain tumor mutational burden (TMB) (Wan et al. 2020). We compared TMB between the two subgroups and determined the association between TMB and survival.

### Evaluation of differences in immune infiltration and checkpoint factor expression

We used the “GSVA” and “GSEABase” packages to conduct ssGSEA, on the basis of which we employed the ESTIMATE algorithm to evaluate immune score, stromal score, estimate score, and tumor purity (Yoshihara et al. 2013). Using the LM22 gene signature matrix downloaded from the CIBERSORT website as a reference (https://cibersortx.stanford.edu/), we determined the relative proportions of 22 immune cell types in each tumor sample (Ren et al. 2021). Finally, the relationship between risk scores, immune scores, immune cell infiltration abundance, and immune checkpoint expression level was analyzed using the “ggplot2” package. Immune function-related genes were collected from TISIDB (an integrated repository portal for tumor-immune system interactions, http://cis.hku.hk/TISIDB/), whereafter the correlation between risk scores and the above genes was analyzed and visualized using the “limma”, “reshape2”, and “RColorBrewer” R packages, to explore the potential mechanism of immune cell infiltration.

### Drug response analysis

IC50 refers to the half-inhibitory concentration of the detected drug and is negatively correlated with the effect of chemotherapeutic drugs. We used the “pRRophetic” package and its functions “car”, “ridge”, “preprocessCore”, “genefilter”, and “sva” to calculate IC50 (Pang et al. 2021). Tumor Immune Dysfunction and Exclusion (TIDE) is a newly developed computational method that identifies the potential for tumor immune escape based on transcriptomic data (Jiang et al. 2018). FPKM gene expression data were Z-score normalized, and TIDE scores were then calculated for BC patients, with high TIDE scores predicting poor ICI efficacy. Accordingly, we analyzed the differences in drug response between patients in high- and low-risk groups, visualizing them via the “ggplot2” package.

### Statistical analysis

All statistical analyses and charts were generated using R software (version 4.2.0), SPSS software (version 18.0), and Excel (Microsoft Corporation, California). The R package in this article is from CRAN (Comprehensive R Archive Network) or Bioconductor (https://www.bioconductor.org/) for download. KM survival analysis and log-rank tests were used to compare survival between high- and low-risk groups. A two-tailed *P*-value less than 0.05 was used as the threshold for statistical significance.

## Results

### BC cases included in the study

The study design is presented in Fig. S1. Mutation analysis included 920 cases, of which 318 were PIK3CA^MUT^, and 602 were PIK3CA^WILD^. Then, 1018 samples with RNA-seq expression data, survival, and clinical information from TCGA BC dataset (n = 1057) and normal (n = 111) (Table S1) were further screened for subsequent analysis (individual clinicopathological factors was not available for some patients, which were included after evaluation). Furthermore, 185 BC samples from GSE48390 (n = 81) and GSE42568 (n = 104) were selected as external cohorts from the GEO database, as per screening criteria, to validate gene signature. The included samples and the corresponding survival information are listed in Table S2.

### Distribution of PIK3CA in BC and its relationship with clinicopathological factors in TCGA and clinical samples

**Fig. 1:**
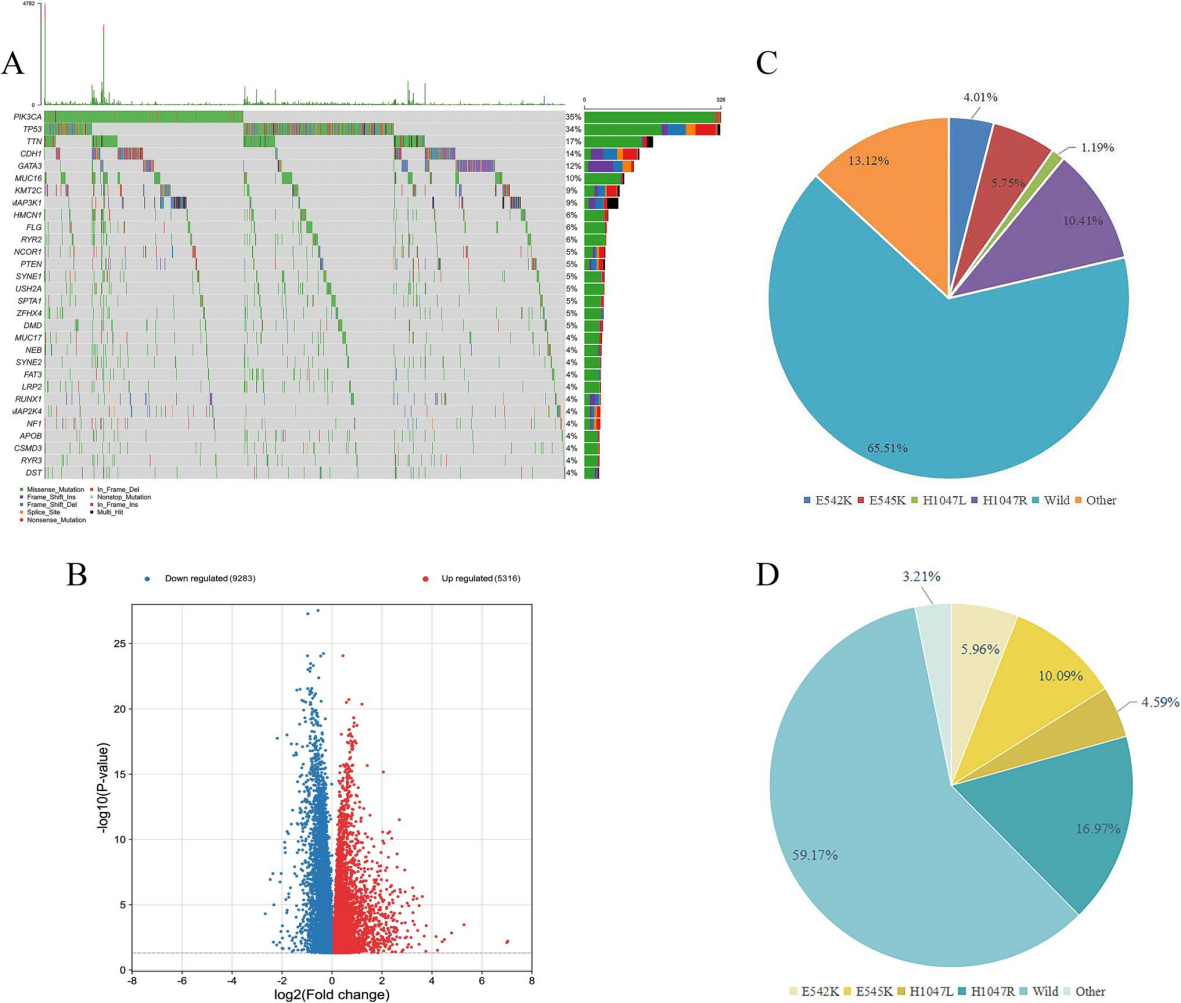
Mutational landscape and distribution of PIK3CA mutation in BC. **A** Frequency and type of mutations in the top 30 genes of BC. **B** Screening for PIK3CA-driven DEGs in PIK3CAMUT and PIK3CAWILD groups by volcano plot. **C** Distribution of PIK3CA mutations in TCGA-BRCA and **D** local hospital cohorts

The waterfall diagram of TCGA cohorts showed that PIK3CA was the most frequently mutated gene in BC, with missense mutations being most common (Fig. 1A). A total of 14,599 DEGs were identified between the PIK3CA^WT^ and PIK3CA^MUT^ groups, of which 5316 and 9283 genes were significantly up- or downregulated in the former, respectively (Fig. 1B). We found that 34.49% (318/ 922) of BC samples harbored PIK3CA mutations (28.30% were exon 9 mutations, 33.65% were exon 20 mutations, and 38.05% were other types of mutations) (Fig. 1C). Of the 218 BC cases tested at the local hospital, 89 patients had PIK3CA mutations (Fig. 1D), accounting for 40.83% (39.33% for exon 9 mutation, 52.81% for exon 20 mutation, 7.86% for other types of mutations). H1047R was the most common mutation type in TCGA and samples from the local hospital, accounting for 30.19% (96/ 318) and 41.57% (37/ 89) of PIK3CA mutations, correspondingly.

We then explored the relationship between PIK3CA mutations and various clinicopathological features. In TCGA database, there was no correlation between PIK3CA mutation status or regions and age, T, N, M, pathological stage, ER, PR, nor HER2 status (Table 1). An association between BC subtypes and PIK3CA mutations was not determined due to insufficient information on molecular subtypes. The number of patients included from our hospital was insufficient to explore the relationship of PIK3CA mutations with metastasis and their impact on prognosis. In our clinical validation cohort, PIK3CA mutation regions were significantly associated with ER (*P* = 0.026) and PR status (*P* < 0.001), but not with other factors (Table 2). It is worth noting that there was a significant correlation between PIK3CA mutation regions and BC subtype (*P* = 0.023), with PIK3CA mutations being more frequent in HR + /HER2- subtype tumors.

**Table 1:** Relationship between PIK3CA mutations and clinicopathological features in TCGA-BRCA cohort

Clinicopathological features	PIK3CA mutation status				PIK3CA mutation regions			
	Mutation	Wild	χ^2^	P	Exon 9	Exon 20	χ^2^	P
	n (%)	n (%)			n (%)	n (%)		
Age (years)								
< 45	40 (12.58)	102 (16.89)	5.807	0.053	11 (10.28)	15 (12.50)	1.879	0.399
45–60	122 (38.36)	252 (41.72)			44 (44.12)	39 (32.50)		
> 60	156 (49.06)	250 (41.39)			52 (48.60)	66 (55.00)		
T classification								
T1–2	276 (86.79)	509 (84.69)	0.737	0.391	93 (86.92)	101 (84.17)	0.344	0.557
T3–4	42 (13.21)	92 (15.31)			14 (13.08)	19 (15.83)		
N classification								
N0	152 (48.25)	271 (46.09)	0.386	0.534	54 (51.43)	62 (51.67)	0.001	0.972
N1–3	163 (51.75)	317 (53.91)			51 (48.57)	58 (48.33)		
M classification								
M0	264 (97.42)	511 (97.52)	0.008	0.931	86 (97.73)	102 (96.23)	/	0.695
M1	7 (2.58)	13 (2.48)			2 (2.27)	4 (3.77)		
Pathological stage								
I–II	243 (78.14)	437 (74.19)	1.712	0.191	86 (80.37)	92 (80.00)	0.005	0.944
III–IV	68 (21.86)	152 (25.81)			21 (19.63)	23 (20.00)		
ER status								
ER negative	70 (22.73)	133 (23.21)	0.026	0.871	27 (26.47)	27 (22.88)	0.381	0.537
ER positive	238 (77.27)	440 (76.79)			75 (73.53)	91 (77.12)		
PR status								
PR negative	106 (34.30)	191 (33.57)	0.049	0.826	40 (39.22)	43 (36.44)	0.179	0.672
PR positive	203 (65.70)	378 (66.43)			62 (60.78)	75 (63.56)		
HER2 status								
HER2 negative	100 (81.30)	171 (74.03)	2.367	0.124	34 (79.07)	40 (85.11)	0.560	0.454
HER2 positive	23 (18.70)	60 (25.97)			9 (20.930	7 (14.89)

**Table 2:** Relationship between PIK3CA mutations and clinicopathological features in local hospital

Clinicopathological features	PIK3CA mutation status				PIK3CA mutation regions			
	Mutation n (%)	Wild n (%)	χ^2^	P	Exon 9 n (%)	Exon 20 n (%)	χ^2^	P
Age(years)								
< 45	18 (20.23)	32 (24.81)	3.430	0.18	11 (30.56)	7 (14.58)	3.133	0.209
45–60	34 (38.20)	59 (45.73)			12 (33.33)	19 (39.58)		
> 60	37 (41.57)	38 (29.46)			13 (36.11)	22 (45.84)		
T classification								
T1–2	87 (97.75)	123 (95.35)	/	0.477	36 (100.00)	46 (95.83)	/	0.504
T3–4	2 (2.25)	6 (4.65)			0 (0)	2 (41.67)		
N classification								
N0	56 (62.92)	75 (58.14)	0.502	0.479	19 (52.78)	34 (70.83)	2.880	0.090
N1–3	33 (37.08)	54 (41.86)			17 (47.22)	14 (29.17)		
Pathological stage								
I–II	76 (85.39)	104 (80.62)	0.834	0.361	28 (77.78)	43 (89.58)	2.192	0.139
III–IV	13 (14.61)	25 (19.38)			8 (22.22)	5 (10.42)		
ER status								
ER negative	23 (25.84)	49 (37.98)	3.510	0.061	5 (13.89)	17 (35.42)	4.932	0.026
ER positive	66 (74.16)	80 (62.02)			31 (86.11)	31 (64.58)		
PR status								
PR negative	33 (37.08)	55 (42.64)	0.676	0.411	6 (16.67)	26 (54.17)	12.267	< 0.001
PR positive	56 (62.92)	74 (57.36)			30 (83.33)	22 (45.83)		
HER2 status								
HER2 negative	65 (73.03)	87 (67.44)	0.780	0.377	30 (83.33)	31 (44.58)	3.637	0.057
HER2 positive	24 (26.97)	42 (32.56)			6 (16.67)	17 (35.42)		
Molecular subtype								
Luminal A	24 (26.97)	27 (20.93)	4.491	0.344	14 (38.89)	7 (14.58)	11.140	0.023
Luminal B (HER2-)	27 (30.34)	41 (31.78)			13 (36.11)	13 (27.08)		
Luminal B (HER2+)	15 (16.85)	16 (12.40)			4 (11.11)	11 (22.92)		
HER2+	9 (10.11)	25 (19.38)			3 (8.33)	6 (12.50)		
TNBC	14 (15.73)	20 (15.51)			2 (5.56)	11 (22.92)		

### Immune subtypes distinguished via consensus clustering

**Fig. 2:**
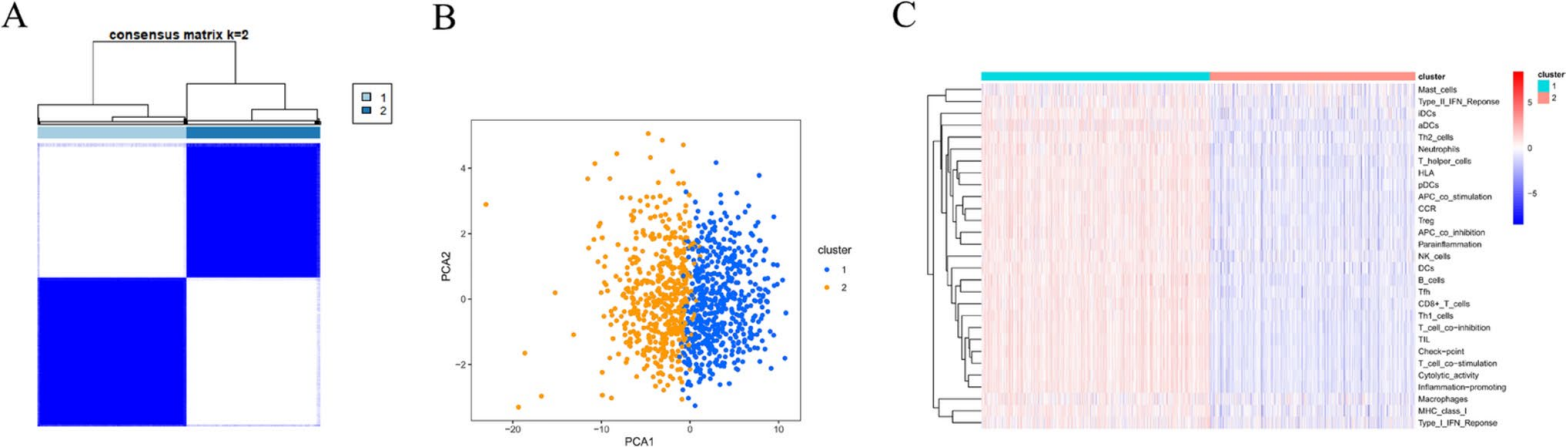
Different immune subtypes distinguished by consensus clustering. **A** The consensus matrix when k = 2. **B** PCA analysis between cluster 1 and cluster 2. **C** The infiltration heatmap of 22 immune cells and 7 immune-related pathways in the two subtypes

Based on the proportion of infiltrating immune cells and pathways, 1057 BC patients were divided into two subgroups (cluster 1 and cluster 2) via consensus clustering (Fig. 2A). PCA analysis revealed that cluster 1 and cluster 2 exhibited obvious separation in spatial distribution (Fig. 2B). The level of immune infiltration was higher in cluster 1 compared to cluster 2, and the former was therefore defined as the high-immunity group (Fig. 2C).

### Screening of co-expression module most relevant to immune subtypes based on WGCNA

**Fig. 3:**
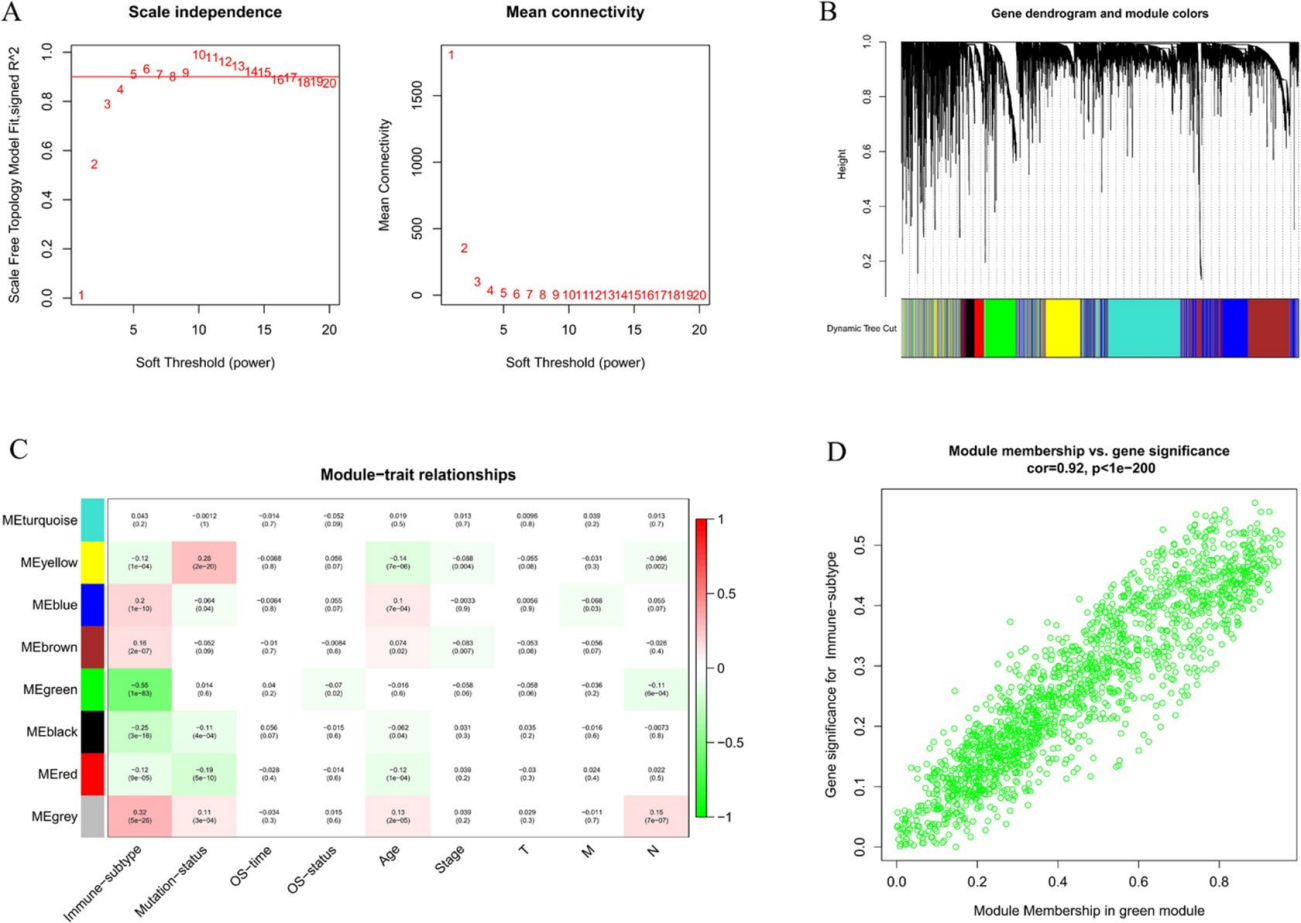
Screening of immune-related genes based on WGCNA. **A** Analysis of the scale-free fit index (left) and the mean connectivity (right) for various soft-thresholding power. **B** The hierarchical clustering dendrogram and the corresponding assigned module colors. **C** Heatmap of correlation between modules obtained by WGCNA and clinical traits. **D** Scatter plot of relevance between genes within the green module and immune subtypes

WGCNA was used to explore the module and hub genes most related to immune subtypes in BC, and β = 5 was selected as the best soft threshold with scale-free R^2^ = 0.9 (Fig. 3A). Eight modules were finally identified when the dynamic shear tree was used to obtain co-expression modules (Fig. 3B). In addition to immune subtype, we included other clinical characteristics (including mutation status, OS time, OS status, age, stage, T, N, M) from TCGA data when performing modular trait correlation analysis. The heatmap indicated that the green module was most associated with immune subtype (ME = − 0.55, *P* < 0.001) and was significantly correlated with OS status (ME = − 0.07, *P* = 0.02) and N stage (ME = − 0.11, *P* < 0.001), including 1635 genes (Fig. 3C). The scatter plot revealed a strong relationship between Gene significance (GS) and Module Membership (MM) (cor = 0.92, *P* < 0.001), indicating that these genes were highly correlated not only with the green module but also with immune subtypes, which warranted further investigation (Fig. 3D).

### Construction of prognostic PDIS in TCGA-BRCA and validation of its predictive ability in GEO cohorts

**Fig. 4:**
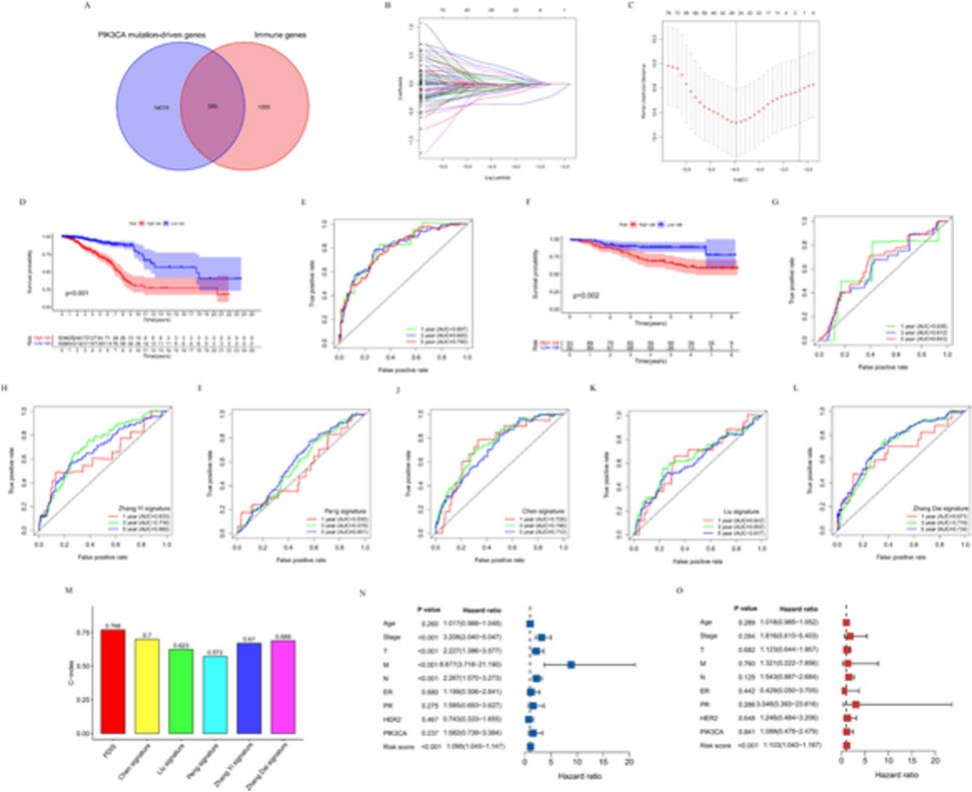
Construction and validation of prognostic PDIS. **A** PDIGs were obtained by Venn diagram. **B** Ten-fold cross-validation for the coefficients. **C** Parameter selection of the 26 selected PDIGs in LASSO regression. **D** KM survival analysis of OS in high- and low-risk patients in TCGA-BRCA cohort. **E** ROC curves of PDIS predicting OS at 1-, 3-, and 5-year in TCGA-BRCA cohort. **F** KM survival analysis in the GEO validation cohorts. **G** ROC curves of PDIS predicting OS in GEO validation cohorts. ROC curves of **H** Zhang Yi signature, **I** Peng signature, **J** Chen signature, **K** Liu signature, and **L** Zhang Dai signature predicting OS at 1-, 3-, and 5-year. **M** Comparison of C-index of predictive power in multiple models. **N** Univariate and **O** multivariate analysis of various factors for OS

Five hundred eighty PDIGs were obtained from the intersection of 14,599 PIK3CA mutation-driven genes and 1635 immune genes in the Venn diagram (Fig. 4A). Univariate Cox regression analysis was performed on the above genes, and 136 genes related to the prognosis of BC patients were obtained (Table S3). LASSO regression analysis was then performed, and the coefficients of 26 genes were retained greater than 0 (Fig. 4B, C). Then multivariate Cox regression analysis was performed on these 26 genes to select independent prognostic genes related to survival, and 16 PDIGs were obtained to construct PDIS, including 11 protective genes and 5 risk genes (Figure S2). The risk score was calculated as: Risk score = h0(t) × e (0.4662 * ADORA3) + (0.5468 * C2CD2) + (−0.2922 * CHST10) + (−0.5043 * DTX1) + (−0.1808 * FKBP5) + (−0.2313 * H1−0) + (−0.1762 * HSPA2) + (−0.2945 * JAK2) + (0.2275 * LINC00992) + (−0.2190 * LYSMD2) + (−0.4286 * NFKBIA) + (−0.3825 * PARP12) + (−0.2276 * PDCD4−AS1) + (0.3791 * PPA2) + (0.2722 * PROM2) + (−0.1232 * TCN1). The patients were divided into high- and low-risk groups based on the median risk score. The KM survival curve indicated that OS in the high-risk group was obviously lower than that in the low-risk group (*P* < 0.001, Fig. 4D). The predicted AUCs of PDIS for 1-, 3-, and 5-year OS were 0.807, 0.800, and 0.790, respectively (Fig. 4E).

GSE48390 and GSE42568 cohorts were used to further validate the predictive ability of PDIS. The risk score of each patient was calculated and the cohorts divided into two groups with respect to the median value. KM survival analysis showed that patients in the high-risk group had a significantly worse OS (*P* < 0.001, Fig. 4F). The AUCs of the PDIS for predicting the 1-, 3-, and 5-year survival in the validation cohort were 0.635, 0.612, and 0.643, respectively (Fig. 4G). Compared with other prognostic models published in recent years, the PDIS exhibited higher AUCs and C-index in predicting BC OS, indicative of its superior predictive performance (Fig. 4H–M). Furthermore, univariate and multivariate Cox analyses of clinical factors, PIK3CA mutation status, and risk scores showed that, except for the PDIS, other factors could not independently predict the prognosis of BC patients (Fig. 4N, O). In view of the significant difference in immune activity between HR + and HR- BC, we analyzed the differences in immune activity between samples with different ER and PR status in the high- and low-risk groups defined by PDIS (Table S4–5). We found that there was no significant difference between ER+ and ER−, PR+ and PR− BC samples in the high- and the low-risk group, indicating that PDIS had similar characteristics in tumors with different HR states (Fig. S3).

### Prognostic value of PIK3CA mutations

We explored survival differences based on PIK3CA mutations in the whole BC, high-, and low-risk groups to test whether PIK3CA mutations could predict PDIS-based risk subgroup prognosis. KM survival analysis indicated that neither PIK3CA mutation status (Fig. S4A–C), nor mutation regions (Fig. S4D–F) could accurately stratify BC patients, which also confirmed the greater prognostic value of the PDIS compared to PIK3CA mutation alone.

### Ability of the nomogram to quantitatively predict prognosis

**Fig. 5:**
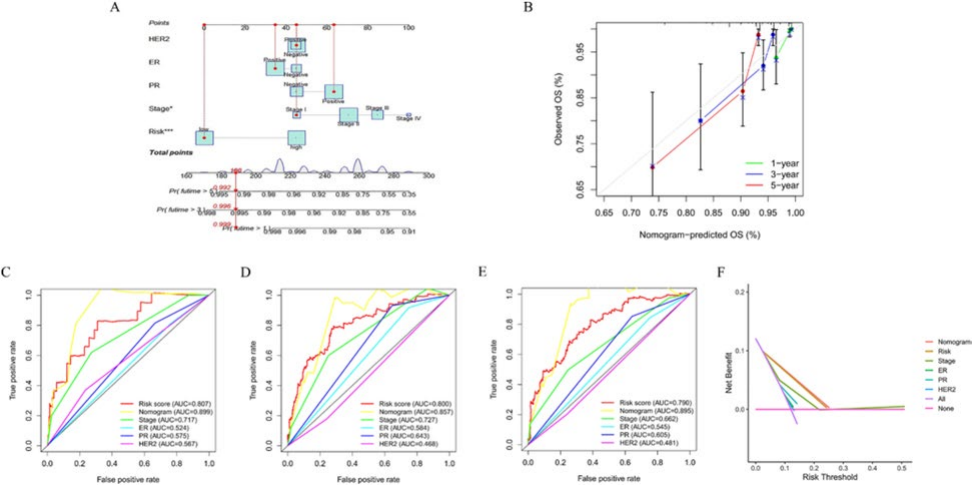
Establishment and verification of predictive nomogram. **A** The total points for each item on the nomogram predicted the survival probability at 3- and 5-year. **B** Calibration curves to assess the consistency between actual survival probability and 1-, 3-, 5-year OS predicted by nomogram. ROC curves of nomogram, PDIS and other prognostic factors for predicting **C** 1-year, **D** 3-year, and **E** 5-year OS. **F** The clinical efficacy of the nomogram, PDIS, and other prognostic factors in predicting 5-year OS was assessed by DCA

Common prognostic predictors (pathological stage, ER, PR, HER2) and PDIS-based risk scores were combined to construct a nomogram to quantitatively predict the OS of BC patients (Fig. 5A). The calibration curve indicated that nomogram-predicted survival was approximately consistent with the actual observed probability, suggesting that the nomogram could accurately predict the 1-, 3-, and 5-year OS (Fig. 5B). Moreover, the predictive ability of the nomogram was higher than that of the PDIS and other predictors based on the AUCs and DCA curves (Fig. 5C–F). In summary, the nomogram can predict BC patient prognosis with greater accuracy.

### Biological function analysis

**Fig. 6:**
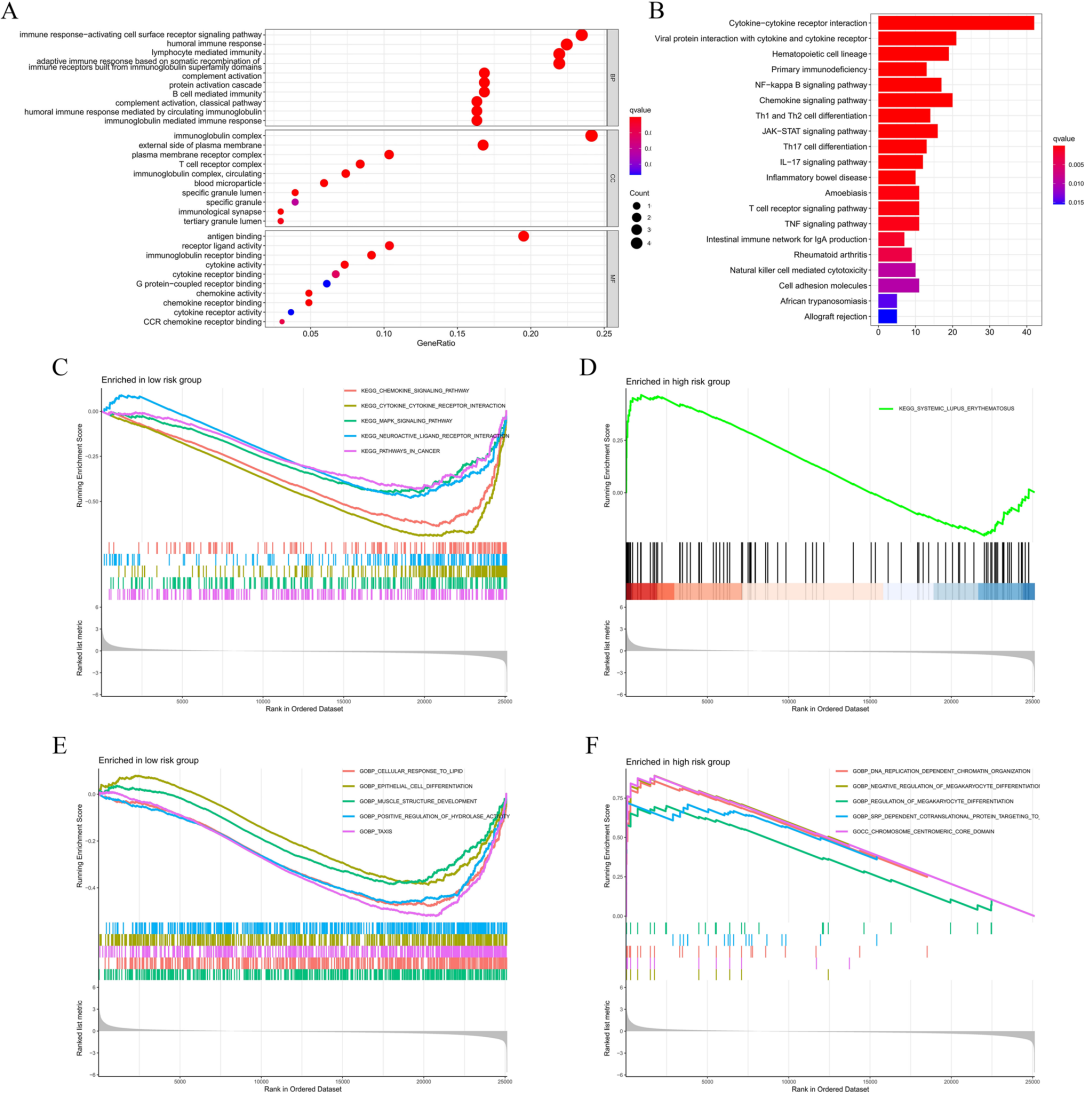
Biological function analysis. **A** GO and **B** KEGG analysis of DEGs between high- and low-risk groups. **C** The top five KEGG enrichment pathways in the low-risk group. **D** The top one KEGG enrichment pathway in the high-risk group. The top five GO enrichment pathways in the **E** low- and **F** high-risk groups

We screened 816 DEGs between PDIS-based risk subgroups, of which 671 were upregulated in the low-risk group, and 145 were upregulated in the high-risk group (Fig. S5). Thereafter, GO and KEGG analysis were employed to explore the biological functions of DEGs. GO analysis revealed that DEGs were significantly enriched in immune-related biological processes, such as immune response-activated cell surface receptor signaling pathway and humoral immune response, among others (Fig. 6A). Similarly, KEGG analysis indicated that DEGs were markedly enriched in a series of signaling pathways (such as cytokine-cytokine receptor interaction, chemokine signaling pathway) that regulate immune, inflammatory, and proliferative processes (Fig. 6B). Subsequently, GSEA was used to determine functional differences between high- and low-risk groups. We found that patients in the high-risk group were enriched in the autoimmunity-related systemic lupus erythematosus pathway, while pathways enriched in the low-risk group were mostly related to chemokines and cytokine-mediated pathways (Fig. 6C, D). Moreover, the two risk groups were significantly associated with the regulation of cell proliferation and differentiation pathway, which is expected to play a role in the development of BC (Fig. 6E, F).

### Mutational landscape of high- and low-risk groups

**Fig. 7:**
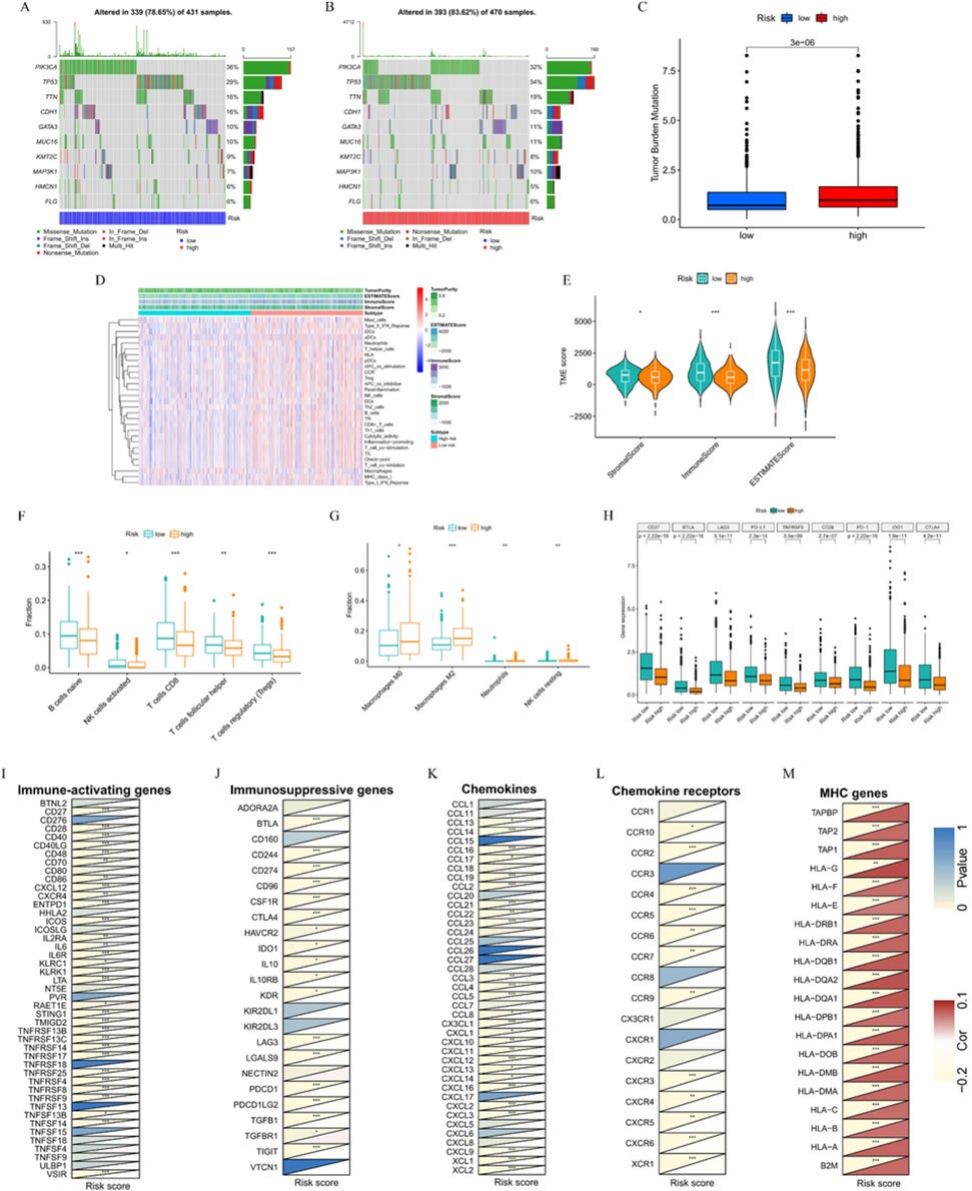
Differences in mutation profiles and immune infiltration between low- and high-risk groups. Frequency and type of mutations in the top 10 genes of **A** low- and **B** high-risk groups. **C** Difference in TMB levels between different risk groups. **D** Infiltration heatmap of 22 immune cell subtypes and 7 immune-related pathways in low- and high-risk groups. **E** Relationship between risk scores and stromal scores, immune scores, and estimated scores. Immune cell types with more infiltration fraction in **F** low- and **G** high-risk groups. **H** The expression levels of nine common immune checkpoint genes in different risk groups. Heatmap of the correlation between PDIS-based risk scores and **I** immune-activating genes, **J** immunosuppressive genes, **K** chemokines, **L** chemokine receptors and MHC genes (*P < 0.05, **P < 0.01, and ***P < 0.001)

The waterfall plots of the top 10 mutated genes in the two risk groups indicated that PIK3CA had the highest mutation frequency in both risk groups, with missense mutations being the most common (Fig. 7A, B). PIK3CA, CDH1, KMT2C, and HMCN1 were more frequently mutated in the low-risk group than in the high-risk group, while the opposite was true for TP53, TTN, GATA3, MUC16, and MAP3K1. The mean TMB value in the high-risk group was significantly higher than that in the low-risk group (Fig. 7C). Taking the median TMB as the cut-off (0.87 mut/MB), KM analysis revealed no significant difference in OS between patients with a high and low TMB (*P* = 0.679).

### Immune infiltration profiles of PDIS-based subgroups

To investigate whether the PDIS reflects the TIME, we analyzed immune cell infiltration and related pathways between high- and low-risk groups. As per the generated heatmap, patients of the low-risk group exhibited greater immune infiltration than those from the high-risk group (Fig. 7D). ESTIMATE analysis also showed that the stromal score, immune score, and ESTIMATE score in the low-risk group were higher than those in the high-risk group (Fig. 7E). Moreover, low-risk tumors harbored greater numbers of naive B cells, activated NK cells, CD8 T cells, follicular helper T cells, and regulatory T cells (Fig. 7F), while M0 and M2 macrophages, neutrophils, as well as resting NK cells were more abundant in the high-risk group (Fig. 7G). These results confirmed that patients in the low-risk group exhibited greater immune cell infiltration. Furthermore, we analyzed the expression of immune checkpoint genes in the two risk subgroups. The expression of PD-L1, PD-1, CTLA4, CD28, CD27, LAG3, IDO1, BTLA, and TNFRSF9 was significantly higher in the low-risk group than in the high-risk group, suggesting that low-risk patients were more likely to benefit from ICIs (Fig. 7H). Notably, most immune-activating genes, immunosuppressive genes, chemokines, and chemokine receptors were negatively correlated with risk scores, while all MHC genes were positively correlated with the risk scores, providing a direction for future studies into the potential mechanisms underlying the observed differences in immune cell infiltration between the high- and low-risk groups (Fig. 7I–M). In addition, PDIS stratification was applied to patients such as TNBC who regularly use ICIs, and different immune activities were observed, low-risk TNBC groups harbored greater numbers of CD8 T cells, regulatory T cells, and M1 macrophages, while M2 macrophages were more abundant in the high-risk TNBC groups (Fig. S6A). The expression of CD27, BTLA and LAG3 in low-risk TNBC patients was significantly higher than that in high-risk group, suggesting that TNBC patients in low-risk group were more likely to benefit from ICI treatment (Fig. S6B).

### Drug sensitivity analysis for immunotherapy, chemotherapy, and targeted therapy

**Fig. 8:**
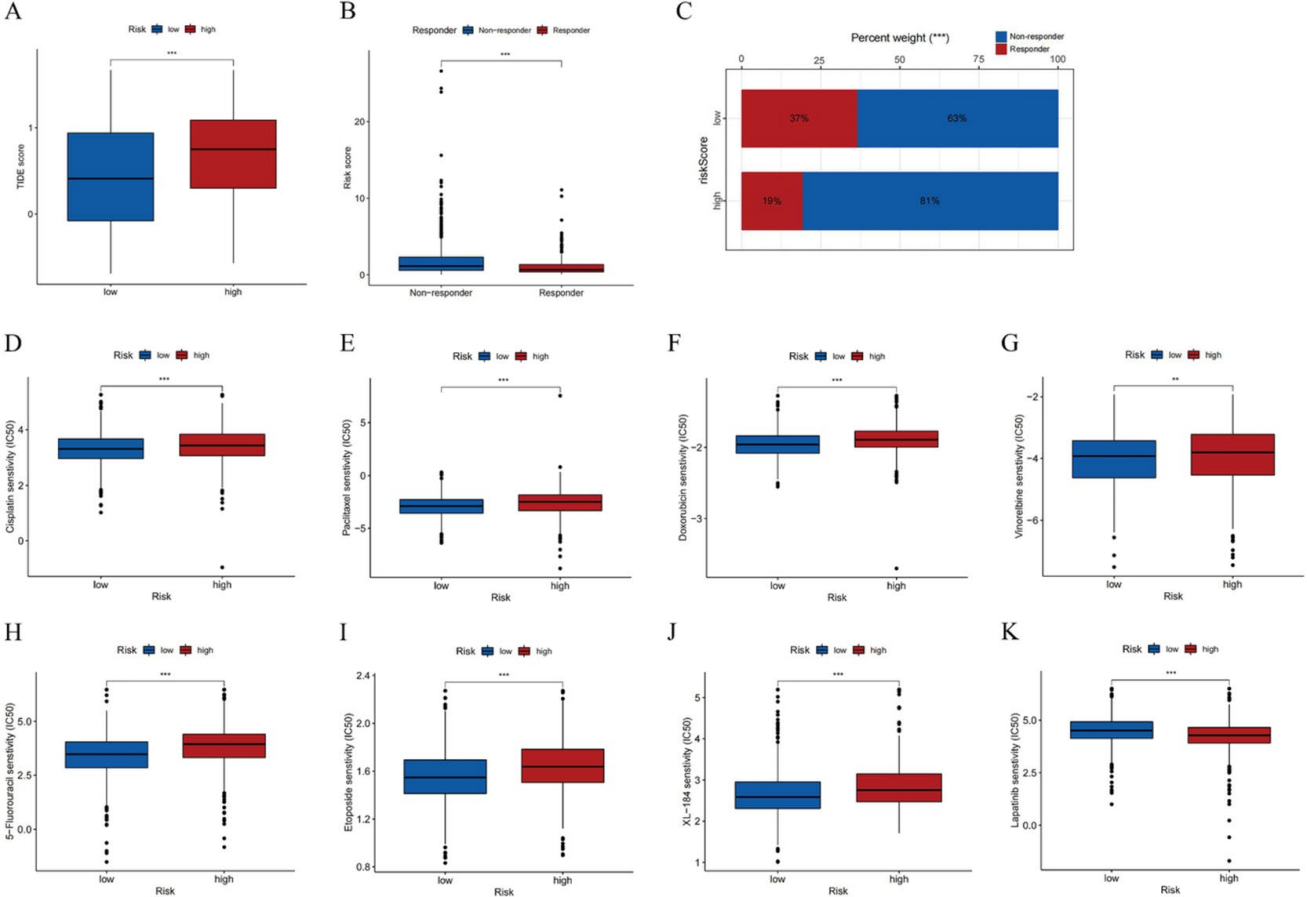
Drug sensitivity analysis of immunotherapy, chemotherapy, and targeted therapies. **A** TIDE scores for different risk groups. **B** Correlation between risk scores and response to immunotherapy. **C** Proportion of non-responder and responder cases to immunotherapy in low- and high-risk groups. The estimated IC50 of some common chemotherapeutic and targeted drugs, such as **D** cisplatin, **E** paclitaxel, **F** doxorubicin, **G** vinorelbine, **H** 5-fluorouracil, **I** etoposide, **J** cabotinib, **K** lapatinib (*P < 0.05, **P < 0.01, and ***P < 0.001)

The response of patients to ICIs in the TCGA-BRCA cohort was predicted by calculating TIDE scores (Table S6). TIDE scores were higher in the high-risk group (Fig. 8A). Based on TIDE scores, patients were divided into responsive and non-responsive groups, and we found that scores in the high-risk group indicated significantly lower efficacy (Fig. 8B, C). This implied that low-risk patients were more likely to benefit from ICI therapy, while the high-risk patients may exhibit greater tumor immune escape and immunotherapy resistance.

According to IC50 levels, risk groups showed differential drug sensitivity to some common chemotherapeutic drugs (cisplatin, paclitaxel, doxorubicin, vinorelbine, 5-fluorouracil, etoposide) and targeted agents (cabotinib, lapatinib). Compared with the high-risk group, patients in the low-risk group were more sensitive to the above-listed chemotherapeutics, suggesting that they may respond better to chemotherapy (Fig. 8D–I). Among targeted agents, low-risk patients were more sensitive to cabotinib (Fig. 8J), while lapatinib was more suitable for high-risk patients (Fig. 8K).

## Discussion

BC is a heterogeneous disease. The molecular subtyping of immunohistochemical markers (ER, PR, HER2, Ki67) has greatly improved prognosis prediction and treatment decision-making (Yeo and Guan 2017). With the development of high-throughput sequencing technology, molecular subtype-based models, such as Oncotype DX, MammaPrint, RecurIndex, Endopredict, and PAM50, have been developed and applied in clinical trials as well as in clinical practice for BC diagnosis, individualized treatment, and survival prediction (Barzaman et al. 2020; Nicolini et al. 2018; Sun et al. 2021). However, these widely used models often neglect the effect of genetic differences on TIME heterogeneity. TIME refers to all immune components within the tumor microenvironment (TME), which plays an established central role in cancer development and progression, having predictive value for BC prognosis and immunotherapy response (Baxevanis et al. 2021; Byrne and Savas 2020; Xu et al. 2021). Tumor genetic heterogeneity, including single-nucleotide variants, short indels, and copy number variants, is involved in establishing TIME heterogeneity (Jia et al. 2022). As the most frequently mutated gene in BC, PIK3CA has gradually become the focus of targeted therapy, but the signature of PIK3CA-driven immune activity driven has not yet been studied.

In the present study, we observed that PIK3CA was most frequently mutated gene in the TCGA-BRCA cohort, with 34.49% samples harboring PIK3CA mutations. H1047R, H1047L, E542K, and E545K accounted for 61.95% of the identified mutations, among which H1047R was the most common, which was basically consistent with the conclusions of other studies (A et al. 2021; Chang et al. 2021; Martínez-Sáez et al. 2020). The clinical relevance of PIK3CA mutations has been extensively studied and is thought to be associated with favorable clinicopathological factors, such as smaller tumor size, HR positivity, and lower grade (Loi, et al. 2013; Sabine et al. 2014; Zardavas et al. 2018). However, no significant association between PIK3CA mutations and clinicopathological features was observed in the TCGA cohort. The characteristics of PIK3CA mutations were validated in clinical samples from a local hospital. The mutation frequency of PIK3CA was 40.83%, with H1047R once again accounting for the highest proportion of mutations. Interestingly, although there remained no link between PIK3CA mutation status and clinicopathological features, we found significant differences in ER, PR status, and molecular subtypes between PIK3CA mutation exon 9 and exon 20, which may provide a basis for individualized endocrine and targeted therapy for patients with different subtypes.

Many previous studies have reported that PIK3CA mutation status may be related to the prognosis of BC patients, but its predictive significance has remained controversial (Baselga et al. 2017; Di Leo et al. 2018; Loi, et al. 2013; Mosele, et al. 2020). Hence, we identified DEGs between PIK3CA^MUT^ and PIK3CA^WILD^ tumors. Two immune subtypes (Immune-H and Immune-L) with differential immune infiltration were distinguished via consensus clustering. The key modules and genes were screened via WGCNA analysis and overlapped with PIK3CA-mutated DEGs to yield PDIGs. After univariate Cox regression, LASSO, and multivariate Cox regression analyses, 16 PDIGs closely related to prognosis were selected to establish PDIS. Validation in the GEO database and comparison with the ROC and C-index of other immune (Chen et al. 2021b; Peng et al. 2021; Zhang et al. 2021), ferroptosis (Liu et al. 2021), and glycolysis-related (Zhang et al. 2020) prognostic models published in recent years demonstrated the prognostic value of the PDIS. Analysis of various prognosis-associated factors revealed that neither PIK3CA mutation status, nor factors commonly employed in clinical practice could independently predict BC prognosis. Meanwhile, the PDIS was an independent predictor of prognosis. Taken together with previous studies, the impact of PIK3CA mutation status or regions on BC prognosis remains largely uncertain to date. Similarly to our work, a number of studies have reported no meaningful association between the two (Loibl et al. 2014; Papaxoinis et al. 2015; Sabine, et al. 2014; Zardavas, et al. 2018). For example, a large research pooling 19 early-stage BC studies revealed that although PIK3CA mutations were associated with better OS in a univariate analysis, this relationship was no longer significant after correction via multivariate analysis, and prognosis was not significantly different between patients carrying mutations in the helical versus kinase domain (Zardavas, et al. 2018). Paradoxically, PIK3CA mutations were reported to predict both a favorable or unfavorable prognosis, varying based on molecular subtype and tumor stage (Deng et al. 2015; Elfgen et al. 2019; Liu et al. 2014; Mosele, et al. 2020; Pang et al. 2014). Still, certain studies suggest biological differences between tumors harboring mutations in the helical or kinase domain, which may be associated with different degrees of invasiveness, yet the specific prognostic value remains undetermined (Barbareschi et al. 2007; Lai et al. 2008; Lerma et al. 2008; Mangone et al. 2012; Pang et al. 2009). In summary, compared with PIK3CA mutation status alone, PDIS exhibits a higher predictive ability for BC patient survival. Moreover, by combining PDIS with ER, PR, HER2 status, and stage, the nomogram constructed can predict the survival probability of patients more accurately and quantitatively than PDIS alone.

Functional analysis revealed that DEGs in the high- and low-risk groups were associated with immune-related biological processes and pathways, as well as that there were differences in the functional pathways involved in the two risk groups that may be associated with BC development. Therefore, we analyzed the immunological profiles of PDIS-based risk groups. The low-risk group possessed a high proportion of infiltrating immunostimulatory cells, such as activated NK cells, CD8 T cells, and follicular helper T cells, which play a role in anti-tumor immunity. Among them, CD8 T cells and follicular helper T cells are important TILs, whose abundant infiltration is widely recognized as a marker of favorable prognosis in BC (Salemme et al. 2021). Conversely, regulatory T cells exhibited relatively abundant infiltration in the low-risk group, exerting an immunosuppressive effect, which has been previously reported as associated with poor prognosis (Martinez et al. 2019; Shash et al. 2021). Immune cells, represented by M2 macrophages, exhibited more abundant infiltration in the high-risk group. Some data suggest that M2 contributes to poor prognosis by stimulating angiogenesis and inflammation, enhancing tumor growth, invasion, and metastasis, whilst promoting immunosuppression (Choi et al. 2018; Mehta et al. 2021). Taken together, it is reasonable to speculate that low-risk patients exhibit a stronger anti-tumor immune response and may respond better to immunotherapy.

ICIs, which block immune checkpoint-mediated suppression to trigger robust anti-tumor immunity, have emerged as one of the most effective forms of immunotherapy, with proven benefit in a variety of cancers (Bagchi et al. 2021; Himmel et al. 2020). However, the low immunogenicity of BC often compromises ICI efficacy, highlighting the need for identifying patients expected to benefit. Herein, we observed a significant increase in the expression of nine common immune checkpoint factors in low-risk patients, including PD-L1, PD-1, and CTLA4, suggesting that this subgroup might benefit from ICIs. It is worth noting that it is recommended to combine PDIS with other indicators commonly used in ICI sensitivity evaluation during BC treatment (including ER, BR, HER2 receptor status, pathological stage, etc.) to play a more valuable role. For example, ICIs are usually used in the treatment of TNBC patients, but the therapeutic effect is limited. TNBC patients stratified by PDIS showed different immune activity. Among the low-risk TNBC population, they may be more likely to benefit from the treatment of ICIs, adding new evidence to the patient’s treatment plan and increasing the patient’s confidence. TMB is considered as a valuable biomarker for predicting ICI response, yet its predictive value varies significantly across cancers (Chan et al. 2019). Our results showed that TMB was generally low in BC, with only 1.8% (17/ 922) exhibiting a TMB greater than 10 mut/Mb in the high-risk group. Further, there was no significant difference in OS between H-TMB and L-TMB, which is consistent with previous findings in BC (McGrail et al. 2021; Wang et al. 2020). H-TMB is associated with a favorable ICI response and prognosis, presumably because mutations give rise to neoantigens that are immunogenic and thus are more likely to trigger T cell responses (Zheng 2022). A threshold of 13 mut/Mb is commonly used in H-TMB cancers, such as melanoma and non-small cell lung cancer. However, as an immunologically “cold” tumor with a low TMB, BC is extremely sensitive to the selection based on a TMB cutoff. In our study, using the median TMB (0.87 mut/Mb) was thus not appropriate. Large prospective studies are needed to determine appropriate cutoff that will allow the effective use of TMB in predicting the response to ICIs and prognosis in BC patients. The value of our PDIS for predicting response immunotherapy and chemotherapy was further explored. The TIDE was reported as an accurate predictor of the response to anti-PD-1 or -CTLA4 when compared to other indicators (e.g., PD-L1 levels and TMB) (Jiang, et al. 2018). We found that TIDE scores were higher in the high-risk group and were associated with poorer ICIs efficacy. The sensitivity of different risk groups to some common chemotherapeutics and targeted therapy drugs varied. Therefore, we have reason to believe that despite the shortcomings of PDIS, it is undeniable that it provides a new potential perspective for the treatment of BC. PDIS is not only an effective biomarker for predicting immunotherapy response, but may also help guide devising chemotherapy regimens in BC patients.

## Conclusions

In the present study, we described PIK3CA mutation characteristics based on TCGA data and cases from a local hospital. The PDIS allowed accurate risk stratification of BC patients with good prognostic predictive power and was validated in the GEO database. The nomogram constructed by combining PDIS and other prognostic factors showed higher clinical efficacy. We also found that the PDIS could assess immune infiltration and help select patients that may benefit from ICIs. Differences in response to immune and chemotherapeutic agents in PDIS-based risk groups suggested that PDIS will be a promising tool to guide clinical treatment decisions and enable individualized therapy.

### Supplementary Information

Below is the link to the electronic supplementary material.

Supplementary file1 (JPG 362 KB)

Supplementary file2 (JPG 805 KB)

Supplementary file3 (JPG 136 KB)

Supplementary file4 (JPG 2352 KB)

Supplementary file5 (JPG 451 KB)

Supplementary file6 (JPG 716 KB)

Supplementary file7 (DOCX 11 KB)

Supplementary file8 (XLS 10 KB)

Supplementary file9 (XLS 16 KB)

Supplementary file10 (XLS 14 KB)

Supplementary file11 (XLS 49 KB)

Supplementary file12 (XLS 49 KB)

Supplementary file13 (XLS 252 KB)

## Data Availability

The data generated and analysed during the current study are available in the TCGA (https://portal.gdc.cancer.gov/) and GEO databases (https://www.ncbi.nlm.nih.gov/geo/).

## Abbreviations

TIME: Tumor immune microenvironment
BC: Breast cancer
TCGA: The Cancer Genome Atlas
PDIG: PIK3CA mutation-driven immune gene
PDIS: PIKC3CA mutation-driven immune signature
TMB: Tumor mutation burden
H-TMB: High tumor mutation burden
L-TMB: Low tumor mutation burden
GEO: Gene Expression Omnibus
PI3K: Phosphatidylinositol 3-kinase
HR: Hormone receptor
ICI: Immune checkpoint inhibitor
CTLA4: Cytotoxic T lymphocyte-associated protein 4
PD-1: Programmed cell death-1
PD-L1: Programmed cell death ligand-1
TIL: Tumor-infiltrating lymphocyte
DEG: Differentially expressed gene
GSEA: Gene set enrichment analysis
ssGSEA: Single-sample gene set enrichment analysis
GO: Gene ontology
KEGG: Kyoto Encyclopedia of Genes and Genomes
