# Supplementary figures and images for: PIK3CA mutation-driven immune signature as a prognostic marker for evaluating the tumor immune microenvironment and therapeutic response in breast cancer
Source: J Cancer Res Clin Oncol. 2024 Mar 11;150(3):119. doi: 10.1007/s00432-024-05626-4 (PMC10927816; doi:10.1007/s00432-024-05626-4)

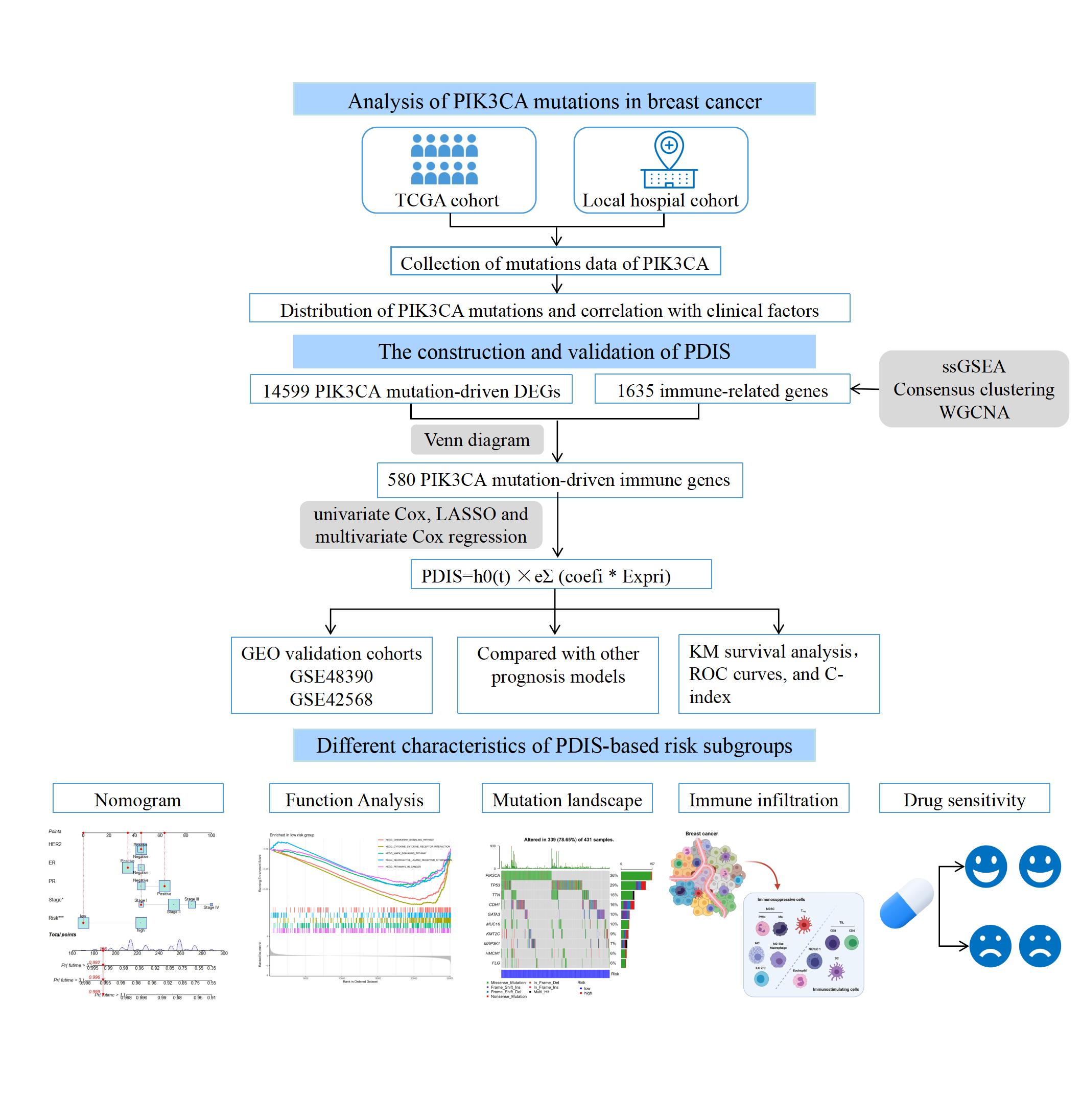

Supplement: Supplementary file 1 — Supplementary file1 (JPG 362 KB) [file 432_2024_5626_MOESM1_ESM.jpg]

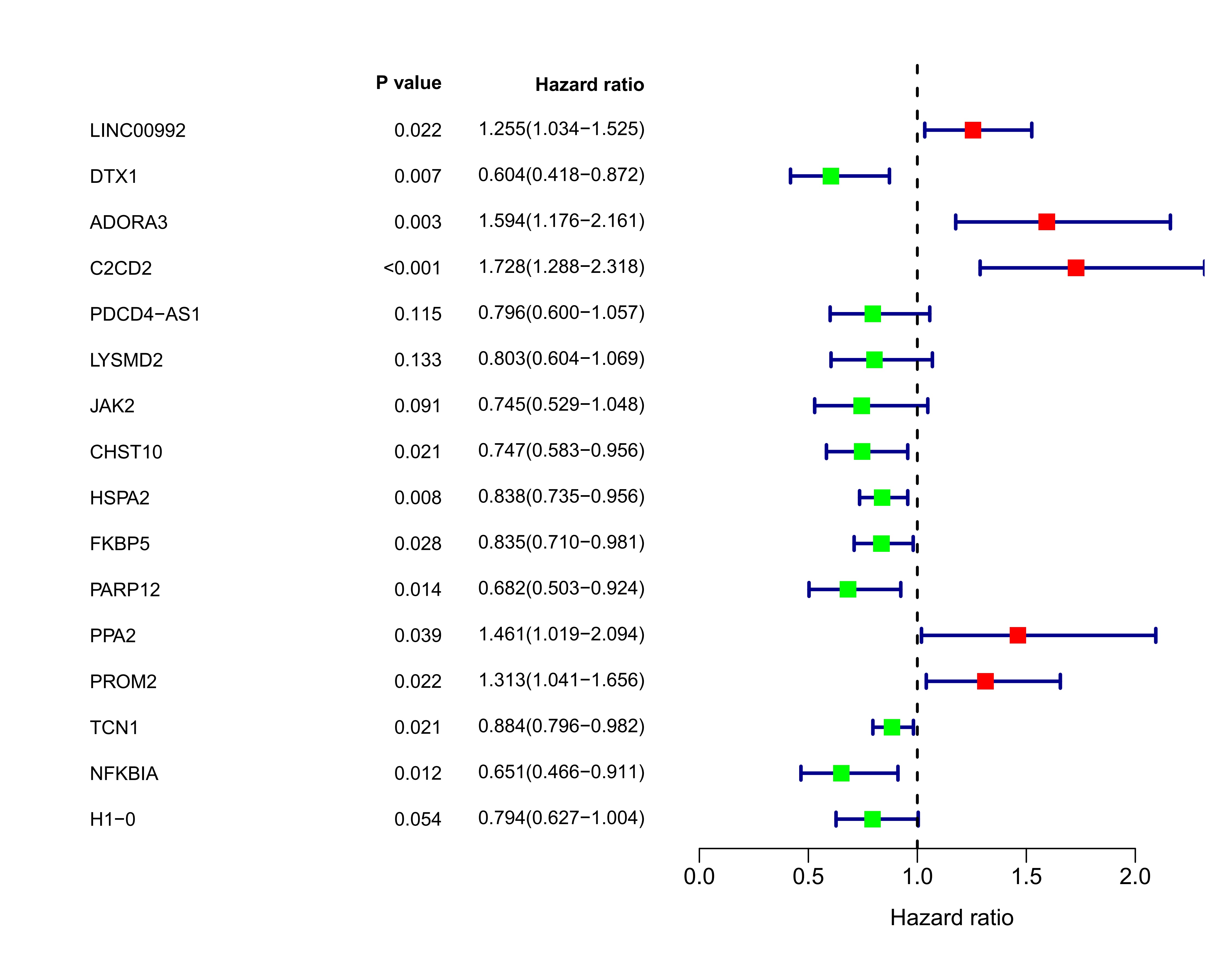

Supplement: Supplementary file 2 — Supplementary file2 (JPG 805 KB) [file 432_2024_5626_MOESM2_ESM.jpg]

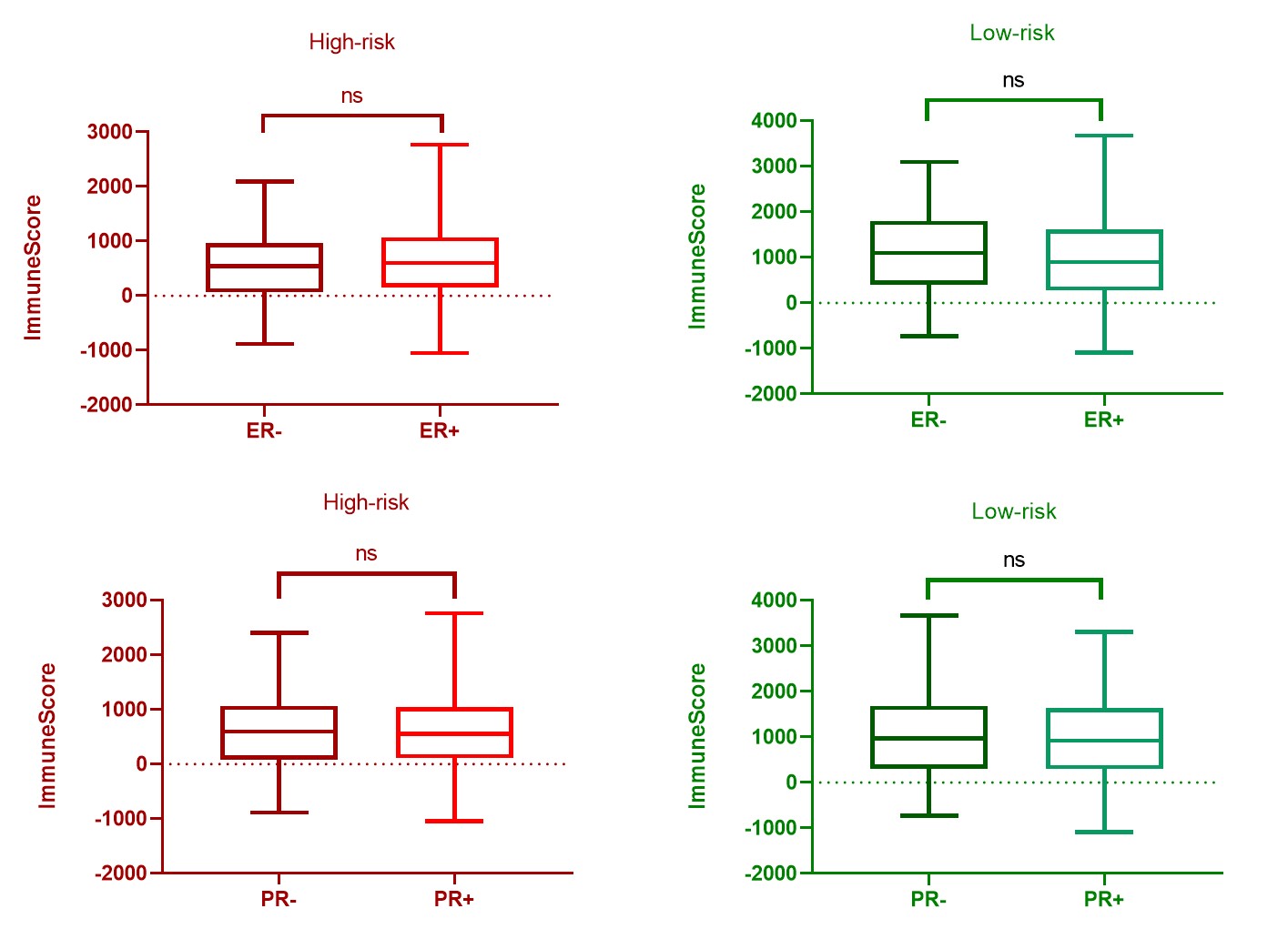

Supplement: Supplementary file 3 — Supplementary file3 (JPG 136 KB) [file 432_2024_5626_MOESM3_ESM.jpg]

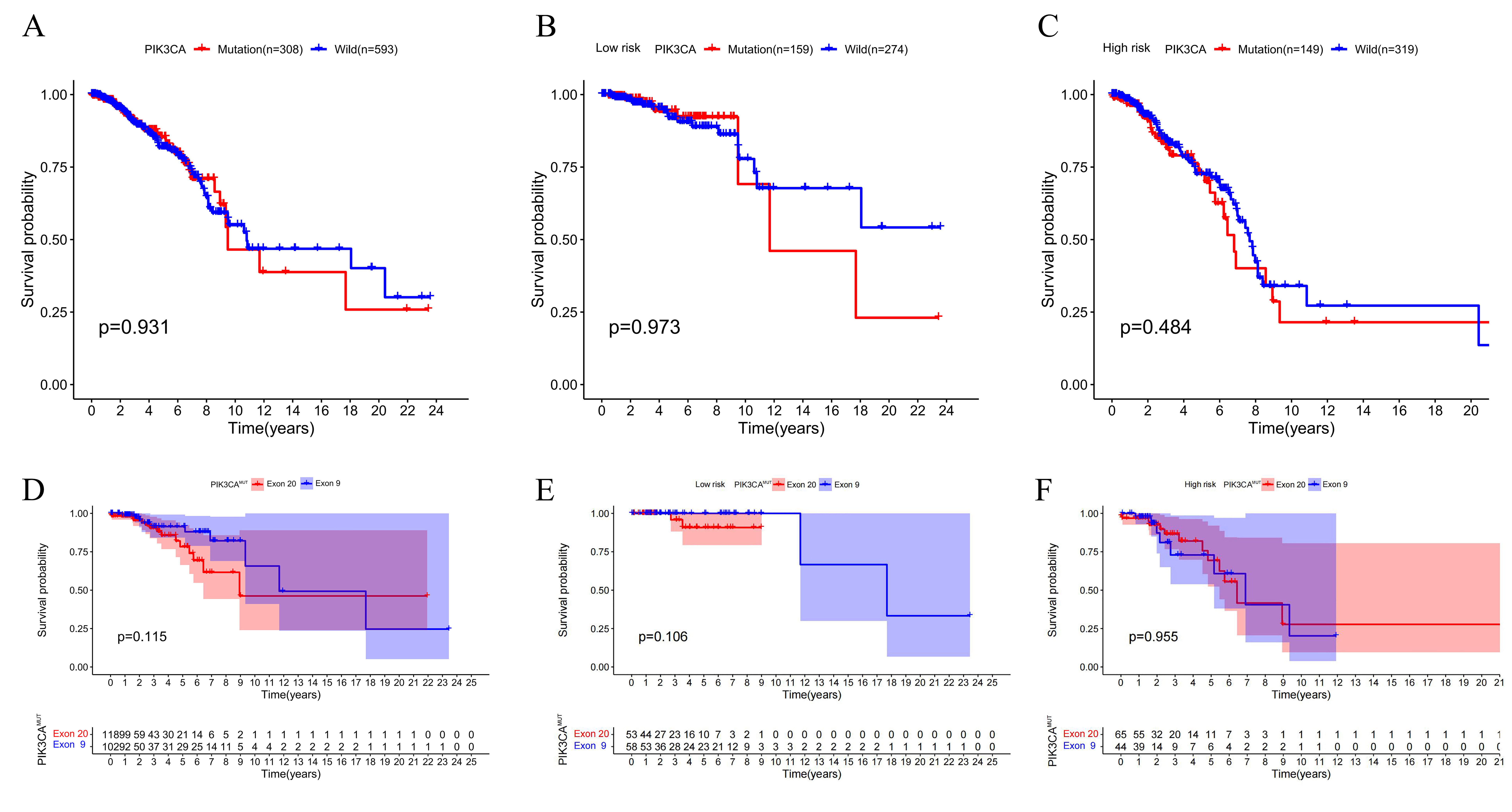

Supplement: Supplementary file 4 — Supplementary file4 (JPG 2352 KB) [file 432_2024_5626_MOESM4_ESM.jpg]

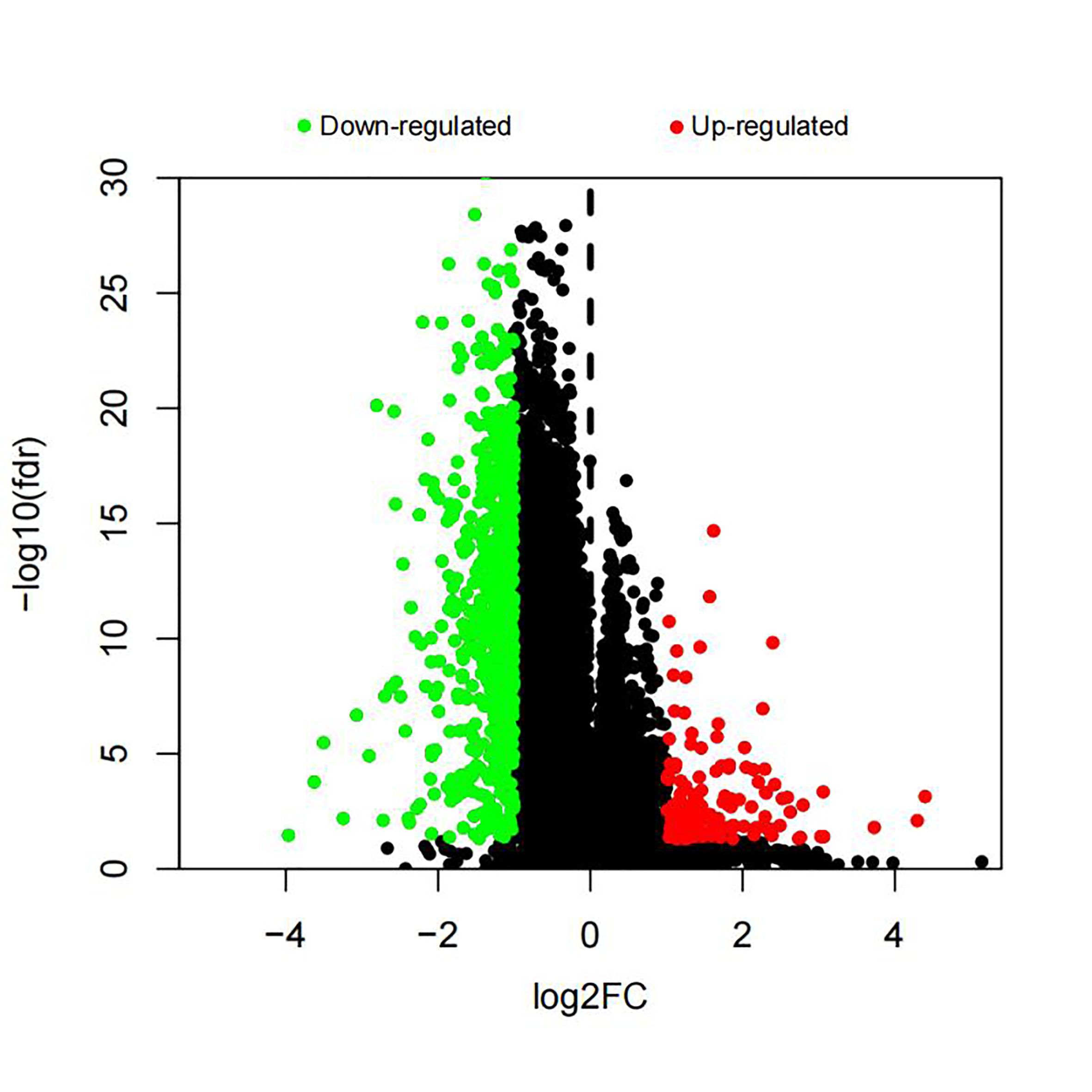

Supplement: Supplementary file 5 — Supplementary file5 (JPG 451 KB) [file 432_2024_5626_MOESM5_ESM.jpg]

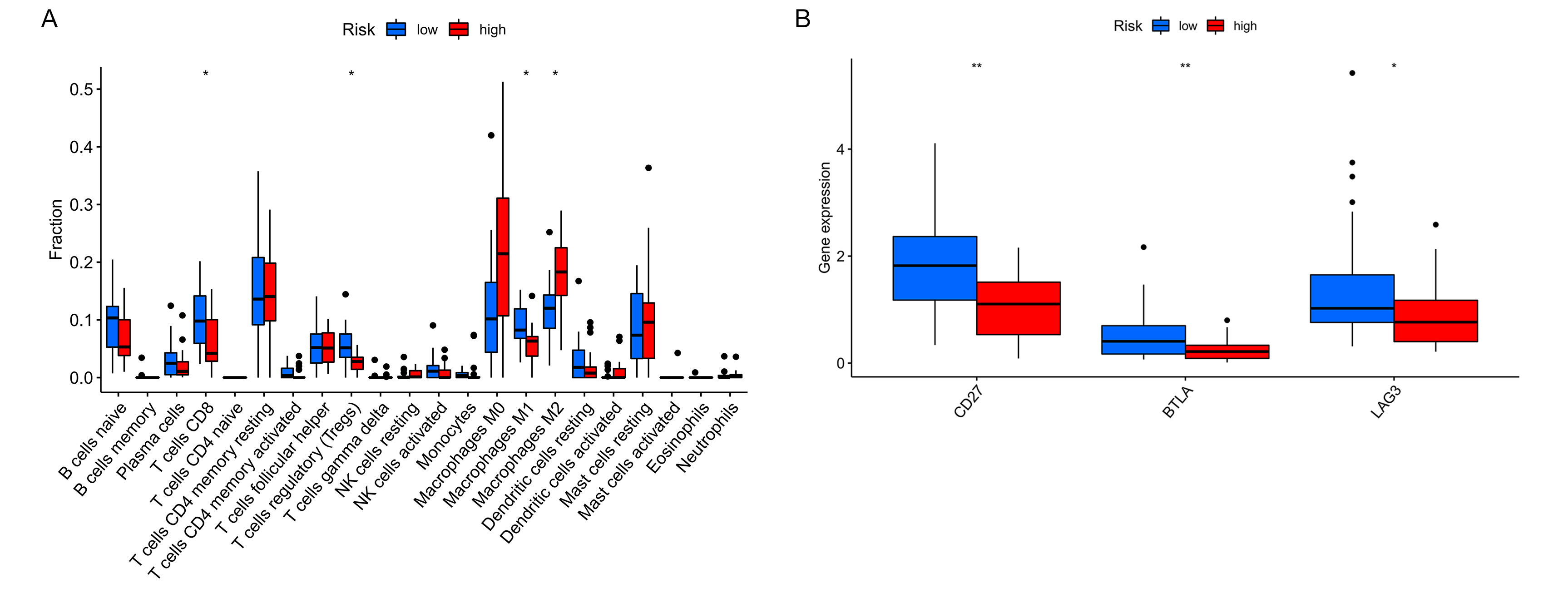

Supplement: Supplementary file 6 — Supplementary file6 (JPG 716 KB) [file 432_2024_5626_MOESM6_ESM.jpg]
