# Supplementary material for: PIK3CA mutation-driven immune signature as a prognostic marker for evaluating the tumor immune microenvironment and therapeutic response in breast cancer
Source: J Cancer Res Clin Oncol. 2024 Mar 11;150(3):119. doi: 10.1007/s00432-024-05626-4 (PMC10927816; doi:10.1007/s00432-024-05626-4)
Supplement: Supplementary file 7 — Supplementary file7 (DOCX 11 KB) [file 432_2024_5626_MOESM7_ESM.docx]

**Figure S1.** Flowchart for the specific analysis of this study.

**Figure S2.** Forest plot of 16 PDIGs used to build PDIS.

**Figure S3.** The relationship between HR status and immune score in the low- and the high-risk groups.

**Figure S4.** Prediction of BC prognosis by PIK3CA mutations. KM survival analysis of PIK3CA^MUT^ and PIK3CA^WILD^ cases in (A) whole BC, (B) low- and (C) high-risk patients. KM survival analysis of exon 9 and exon 20 cases in (D) whole BC, (E) low- and (F) high-risk patients.

**Figure S5.** Volcano plot of DEGs between different risk groups based on PDIS.

**Figure S6.** Differences in immune infiltration between low- and high-risk TNBC groups. (A) The fraction of immune cell in different risk TNBC groups. (B) The expression levels of immune checkpoint genes in different risk TNBC groups. (*P < 0.05, **P < 0.01, and ***P < 0.001)
